# Revolutionizing cancer treatment: enhancing CAR-T cell therapy with CRISPR/Cas9 gene editing technology

**DOI:** 10.3389/fimmu.2024.1354825

**Published:** 2024-02-21

**Authors:** Ruiyu Tao, Xiaopeng Han, Xue Bai, Jianping Yu, Youwei Ma, Weikai Chen, Dawei Zhang, Zhengkai Li

**Affiliations:** ^1^ Department of Gastrointestinal Surgery, Gansu Provincial Maternity and Child-care Hospital, Lanzhou, Gansu, China; ^2^ Department of Urology, Gansu Provincial Maternity and Child-care Hospital, Lanzhou, Gansu, China

**Keywords:** CAR-T cell, CRISPR/Cas9, immune checkpoints, durability, cost, security

## Abstract

CAR-T cell therapy, a novel immunotherapy, has made significant breakthroughs in clinical practice, particularly in treating B-cell-associated leukemia and lymphoma. However, it still faces challenges such as poor persistence, limited proliferation capacity, high manufacturing costs, and suboptimal efficacy. CRISPR/Cas system, an efficient and simple method for precise gene editing, offers new possibilities for optimizing CAR-T cells. It can increase the function of CAR-T cells and reduce manufacturing costs. The combination of CRISPR/Cas9 technology and CAR-T cell therapy may promote the development of this therapy and provide more effective and personalized treatment for cancer patients. Meanwhile, the safety issues surrounding the application of this technology in CAR-T cells require further research and evaluation. Future research should focus on improving the accuracy and safety of CRISPR/Cas9 technology to facilitate the better development and application of CAR-T cell therapy. This review focuses on the application of CRISPR/Cas9 technology in CAR-T cell therapy, including eliminating the inhibitory effect of immune checkpoints, enhancing the ability of CAR-T cells to resist exhaustion, assisting in the construction of universal CAR-T cells, reducing the manufacturing costs of CAR-T cells, and the security problems faced. The objective is to show the revolutionary role of CRISPR/Cas9 technology in CAR-T cell therapy for researchers.

## 1 Introduction

**Figure 1:**
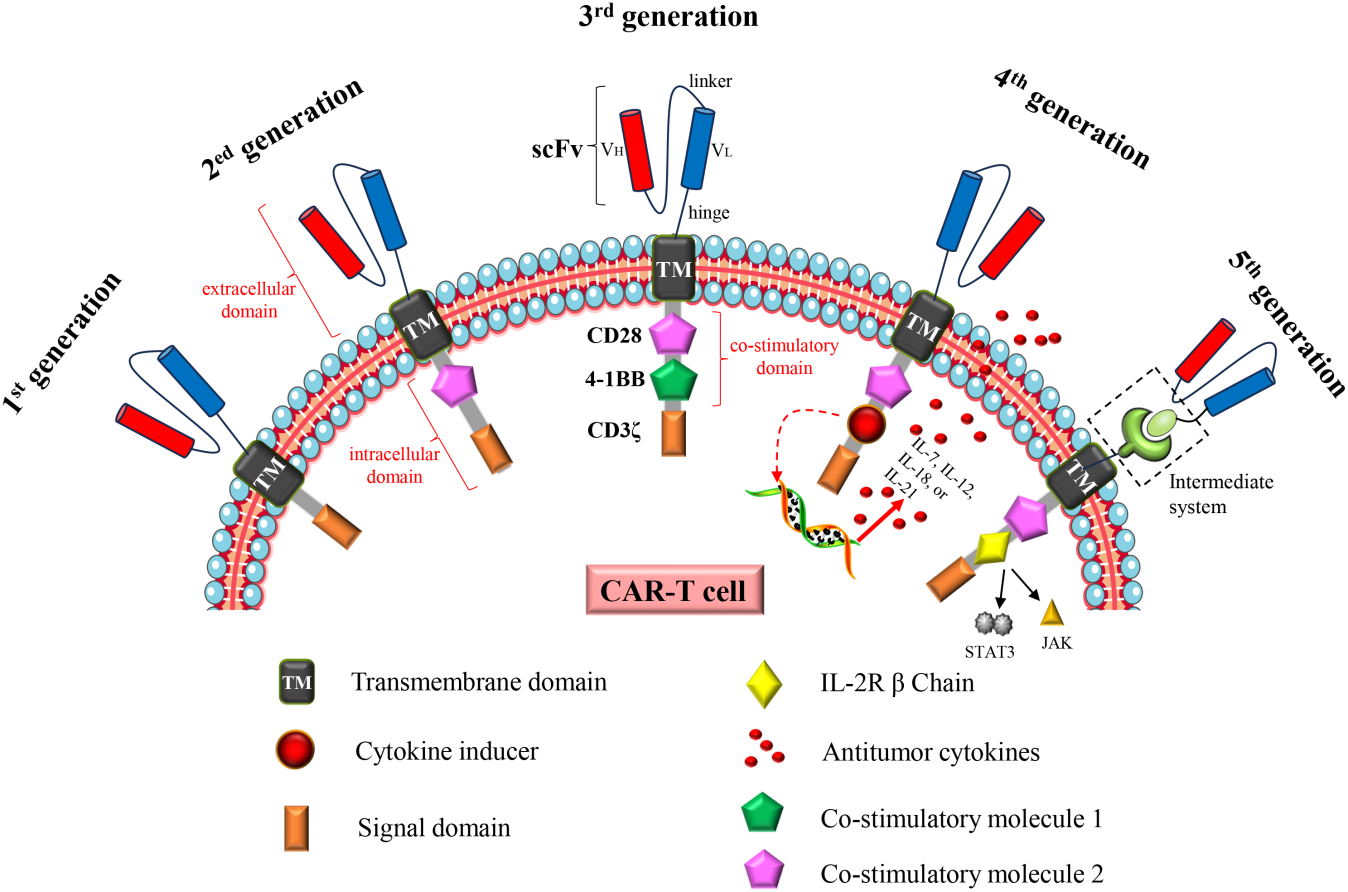
Structural changes to CAR constructs from first to fifth generation. The first generation CAR consists of a single-chain variable fragment (scFv), a transmembrane domain (TM) and an intracellular signaling domain (CD3ζ). The second generation CAR adds a costimulatory molecule (CD28 or 4-1BB) to the intracellular domain. The third generation CAR adds two costimulatory molecules (CD28 and 4-1BB) to the intracellular domain; The fourth generation CAR is based on the second generation CAR or the third generation CAR, and the cytokine receptor genes are added to the intracellular domain. The fifth generation CAR is to add an intermediate system in the extracellular domain, separating the scFv from the transmembrane domain in the extracellular domain; or insert an IL2 RB chain fragment between the costimulatory domain (CD28) and CD3ζ, inducing activation of the JAK-STAT pathway, thereby promoting cell proliferation. In addition, universal CAR-T cells generated using universal T cells (TCR gene and/or HLA class I locus of allogeneic T cells have been destroyed) also belong to the fifth generation CAR-T cells.

The increasing incidence and mortality of cancer have become a major global problem, necessitating the expeditious development of efficacious therapeutic drugs or methodologies. Chimeric antigen receptor (CAR)-T cell therapy opens up a new road for cancer treatment. The principle is to engineer patients’ T cells *in vitro* to express specific CAR proteins on the T cell surface. These T cells expressing CAR proteins can recognize and target the corresponding tumor antigens, and ultimately kill tumor cells. The concept of “CAR” was first proposed in 1989 (1, 2), and it has developed into the fifth generation CAR after more than two decades of development (**Figure 1**). There has been a rapid advancement in CAR-T cell therapy since 2012 (3). Up to now, several CAR-T cell therapies for hematological malignancies have been marketed, and the related targets include CD19 and B cell mature antigen (BCMA). Furthermore, a substantial number of clinical trials pertaining to this therapy continue to be conducted and registered annually. Over 200 studies related to CAR-T cell therapy were registered in 2023 (ClinicalTrials.gov, last accessed on December 3, 2023).

Despite the efficacy of the therapy in certain hematological malignancies, several challenges persist, including issues related to persistence, drug resistance, immune evasion, and toxic reactions (4–7). Additionally, CAR-T cells have limited effectiveness in treating solid tumors. Consequently, researchers have endeavored to enhance both the efficacy and safety of the therapy by optimizing CAR structure or employing combination therapies (8, 9), such as combined radiotherapy (10), chemotherapy (11), immune checkpoint inhibitor (12), and oncolytic virus (13). These strategies have demonstrated some improvement in the effectiveness and safety of CAR-T cells; however, they have not fully resolved the aforementioned issues. Moreover, the considerable expense associated with this therapy has somewhat impeded its widespread adoption in clinical settings (14). Hence, there remains a need to further refine CAR-T cells to improve their effectiveness and safety, while also reducing the costs associated with their manufacturing.

The clustered regularly interspaced short palindromic repeat (CRISPR)/Cas9 technology, a groundbreaking gene editing tool, has gained significant traction in the field of life science research. In 2012, Jennifer Doudna and Emmanuelle Charpentier first proved the potential of CRISPR/Cas9 in gene editing in prokaryotes (15). Subsequently, Zhang Feng et al. (16) demonstrated for the first time that CRISPR/Cas9 technology can also perform gene editing in human cells or mammalian cells. This technology utilizes the CRISPR/Cas system found in the natural immune system of bacteria and achieves precise gene cutting and editing through the Cas9 protein and guide RNA. It offers a simple, efficient, and cost-effective approach to editing almost any gene. Recently, researchers have started to optimize and modify CAR-T cells using CRISPR/Cas9 technology to enhance their targeting, persistence, antitumor activity, and safety. Exploring the application of this technology in CAR-T cells has become a prominent research focus in the field (17).

## 2 CRISPR/Cas9 technology and pCAR-T cells

Taking advantage of CAR-T cells for cancer treatment is a promising strategy, particularly for patients who have not responded to traditional therapies. However, this therapy also faces some urgent problems. Addressing these challenges and optimizing CAR-T cells to enhance their therapeutic effectiveness and safety is a crucial area of research in this field. CRISPR/Cas9 technology has made designing and optimizing CAR-T cells easier and more convenient. In recent years, the application of CRISPR/Cas9 technology in the research of CAR-T cells has been steadily increasing. At present, several clinical trials of engineered CAR-T cells based on CRISPR/Cas9 technology are underway or have been completed, see **Table 1**. These trials highlight the immense potential of utilizing this technology to optimize and improve CAR-T cells.

**Table 1:** Clinical trials of engineering CAR-T cells based on CRISPR/Cas9 technology.

Study Title	Phase	CAR-T Cell	Cancer	Edited/targeted Genes	Patients(n)	Location	Status	NCT Number
CRISPR-Edited Allogeneic Anti-CD19 CAR-T Cell Therapy for Relapsed/Refractory B Cell Non-Hodgkin Lymphoma (ANTLER)	I	CD19-CAR-T	NHL	Unknown	72	United States	Recruiting	NCT04637763
A Safety and Efficacy Study Evaluating CTX130 in Subjects with Relapsed or Refractory T or B Cell Malignancies (COBALT-LYM)	I	CD70-CAR-T	T cell malignancy, DLBCL	Unknown	45	United States	Recruiting	NCT04502446
A Safety and Efficacy Study Evaluating CTX120 in Subjects with Relapsed or Refractory Multiple Myeloma	I	BCMA-CAR-T	MM	Unknown	26	United States	Active, not recruiting	NCT04244656
CRISPR-Edited Allogeneic Anti-BCMA CAR-T Cell Therapy in Patients with Relapsed/Refractory Multiple Myeloma	I	BCMA-CAR-T	MM	Unknown	50	United States	Recruiting	NCT05722418
A Safety and Efficacy Study Evaluating CTX130 in Subjects with Relapsed or Refractory Renal Cell Carcinoma (COBALT-RCC)	I	CD70-CAR-T	Renal Cell Carcinoma	Unknown	107	United States	Active, not recruiting	NCT04438083
A Safety and Efficacy Study Evaluating CTX110 in Subjects with Relapsed or Refractory B-Cell Malignancies (CARBON)	I/II	CD19-CAR-T	B-cell Lymphoma, B-cell ALL	Unknown	227	United States	Recruiting	NCT04035434
A Safety and Efficacy Study Evaluating CTX131 in Adult Subjects with Relapsed or Refractory Solid Tumors	I/II	CD70-CAR-T	Multiple Solid Tumors	Unknown	250	United States	Recruiting	NCT05795595
A Safety and Efficacy Study Evaluating CTX112 in Subjects with Relapsed or Refractory B-Cell Malignancies	I/II	CD19-CAR-T	Leukemia, CLL	Unknown	120	United States	Recruiting	NCT05643742
CRISPR-Edited Allogeneic Anti-CLL-1 CAR-T Cell Therapy in Patients with Relapsed/Refractory Acute Myeloid Leukemia	I	CLL-1-CAR-T	AML	Unknown	70	-	Not yet recruiting	NCT06128044
Programmed Allogeneic CRISPR-edited T Cells Engineered to Express Anti-CD19 Chimeric Antigen Receptor (PACE CART19) in Patients with Relapsed Or Refractory CD19+ Leukemia and Lymphoma	I	CD19-CAR-T	ALL, CLL, NHL	B2M, CIITA, and TCR alpha chain (knock out)	–	–	Withdrawn	NCT05037669
Study of CRISPR-Cas9 Mediated PD-1 and TCR Gene-knocked Out Mesothelin-directed CAR-T Cells in Patients with Mesothelin Positive Multiple Solid Tumors.	I	Mesothelin-CAR-T	Multiple Solid Tumors	PD-1 and TCR (knock out)	10	China	Unknown status	NCT03545815
Study of PD-1 Gene-knocked Out Mesothelin-directed CAR-T Cells with the Conditioning of PC in Mesothelin Positive Multiple Solid Tumors	I	Mesothelin-CAR-T	Multiple Solid Tumors	PD-1 (knock out)	10	China	Unknown status	NCT03747965
PD-1 Knockout Anti-MUC1 CAR-T Cells in the Treatment of Advanced Breast Cancer	I/II	MUC1-CAR-T	Breast Cancer	PD-1 (knock out)	15	China	Completed	NCT05812326
CRISPR (HPK1) Edited CD19-specific CAR-T Cells (XYF19 CAR-T Cells) for CD19+ Leukemia or Lymphoma.	I	CD19-CAR-T	ALL, Lymphoma	HPK1 (knock out)	40	China	Recruiting	NCT04037566
A Feasibility and Safety Study of Universal Dual Specificity CD19 and CD20 or CD22 CAR-T Cell Immunotherapy for Relapsed or Refractory Leukemia and Lymphoma	I/II	CD19-CD20-CAR-T, CD22-CAR-T	Leukemia, Lymphoma	Unknown	80	China	Unknown status	NCT03398967
A Study Evaluating UCART019 in Patients with Relapsed or Refractory CD19+ Leukemia and Lymphoma	I/II	CD19-CAR-T	Leukemia, Lymphoma	TCR and B2M (knock out)	80	China	Unknown status	NCT03166878
TT52CAR19 Therapy for B-cell Acute Lymphoblastic Leukaemia (B-ALL)	I	CD19-CAR-T	ALL	CD52 and TRAC loci (knock out)	10	United Kingdom	Active, not recruiting	NCT04557436

Data extracted from https://clinicaltrials.gov/. NHL, Non-Hodgkin Lymphoma; ALL, Acute Lymphocytic Leukemia; DLBCL, Diffuse Large B-Cell Lymphoma; MM, Multiple Myeloma; CLL, Chronic Lymphocytic Leukemia; AML, Acute Myeloid Leukemia; CLL-1, C-type lectin-like molecule-1; MUC-1, mucin-1; B2M, Beta-2 microglobulin; TCR, T cell receptor; HPK1, Hematopoietic Progenitor Kinase 1.

### 2.1 CRISPR/Cas9 technology can optimize CAR-T cell function

#### 2.1.1 Eliminating the inhibition of immune checkpoints

Immune checkpoints play a crucial role as regulatory molecules within the immune system, exerting a negative influence on immune cell activation and impeding immune cell activity to prevent autoimmune overreaction. On the other hand, this mechanism also enables cancer cells to evade immune attacks. Among them, PD-1 and CTLA-4 are the most deeply studied immune checkpoint molecules. By obstructing the binding between immune checkpoints and their respective ligands, immune checkpoint inhibitors can effectively restore T cell immune function. To mitigate the immune checkpoint inhibition of CAR-T cells, a research team sought to enhance the efficacy of cancer treatment by combining PD-1 inhibitors with CAR-T cells. Specifically, the team engineered Mesothelin-CAR-T cells for the purpose of treating pleural mesothelioma in mice, and administered them in conjunction with a PD-1 inhibitor. The findings demonstrated that this combined therapeutic approach effectively extended the duration of CAR-T cell activity, significantly impeded tumor progression, and significantly prolonged the median survival time (18). Subsequently, the research team successfully validated the feasibility of employing CAR-T cells in combination with PD-1 inhibitors for treating malignant pleural mesothelioma in one clinical trial (19). Given that blocking immune checkpoints has the potential to reactivate T cells, a key question of interest would be whether selective reduction or elimination of immune checkpoint genes on T cells effectively maintain or enhance CAR-T cell function? Research has proved that employing CRISPR/Cas9 to delete PD-1 resulted in enhanced long-term persistence and activity of CAR-T cells (preclinical study) (20), and similarly, the deletion of CTLA-4 using CRISPR/Cas9 improved the proliferation and activity of CAR-T cells (preclinical study) (21). Numerous investigations conducted thus far have demonstrated that the abrogation of immune checkpoint inhibition can effectively enhance and optimize the performance of CAR-T cells across various dimensions (**Table 2**). Furthermore, this table presents the associated research pertaining to the manipulation of specific genes through the utilization of CRISPR/Cas9 technology, encompassing both gene knockout and gene knock-in approaches.

Table 2CRISPR/CAS9-based knockout of immune checkpoint and other genes to improve CAR-T cell function.

**Table:** 

Year	Target	Associated tumors	Associated immune checkpoints	Method	Consequence
2023	CD19	-	PD-1	CRISPR/Cas9-mediated PD-1 knockout	Improved the durability of CAR-T cells (20)
2023	CD19	ALL, MM	CTLA-4	CRISPR/Cas9-mediated CTLA-4 knockout	Increased the proliferation and antitumor activity CAR-T cells (21)
2022	CD19	NHL	PD-1	CRISPR/Cas9-mediated destruction of PD-1	Can produce more memory T cells and enhance the antitumor ability (22)
2021	CD19	bladder cancer, melanoma, lymphocytic leukemia	SOCS1	CRISPR/Cas9-mediated SOCS1 knockout	SOCS1 deletion enhanced CD4+T cell proliferation, persistence, and effector function, while SOCS1 deletion only enhanced CD8+T cell survival and effector function (23)
2021	MSLN	MSLN^+^ solid tumors	PD-1	CRISPR/Cas9-mediated PD-1 knockout	It was preliminarily proved that PD-1 deficient CAR-T cells were safe and feasible in human body (24)
2017	CD19, PSCA	–	PD-1	CRISPR/Cas9-mediated TCR, HLA class I molecule, and PD-1 knockout	Reduced the level of allogeneic reaction of CAR-T cells, and improved the anti-tumor activity (25)
2017	CD19	Hematologic malignancy	LAG-3	CRISPR/Cas9-mediated LAG-3 knockout	LAG-3 deficient CAR-T cells had comparable antitumor activity to normal CAR-T cells (26)
2019	MSLN	TNBC	PD-1	CRISPR/Cas9-mediated PD-1 knockout	Enhanced the antitumor effect of CAR-T cells (27)
2019	EGFRvIII	glioblastoma	PD-1	CRISPR/Cas9-mediated TRAC, B2M, and PD-1 knockout	Reduced the level of allogeneic reaction of CAR-T cells, and improved the antitumor activity (28)
2020	EGFRvIII	glioblastoma	PD-1	CRISPR/Cas9-mediated PD-1 knockout	Enhanced the antitumor effect of CAR-T cells (29)

**Table:** 

Year	Target	Associated tumors	Other genes	Method	Consequence
**2017**	CD19	ALL	TRAC	Using CRISPR/Cas9 technology to direct CAR gene to the TRAC locus	Delaying effector T-cell differentiation and exhaustion (30)
**2017**	CD19	leukaemia	Fas receptor gene	Using CRISPR/Cas9 technology to destroy fas receptor gene	Disruption of Fas receptor/Fas ligand interactions to prevent activation induced cell death (31)
**2018**	EGFRvIII	glioblastoma	DGK	CRISPR/Cas9-mediated DGK knockout	Enhancing the proliferation ability and infiltration ability of CAR-T cells (32)
**2019**	CD19	leukaemia	GM-CSF	CRISPR/Cas9-mediated GM-CSF knockout	Improving the anti-tumor effect of CAR-T cells and may reduce the incidence of CRS and neurotoxicity (33)
**2020**	CD19	leukaemia	IL-6	CRISPR/Cas9-mediated IL-6 knockout	May decrease the severity or incidence of sCRS and related complications (34)
**2022**	CD19	ALL	CD52	CRISPR/Cas9-mediated CD52 knockout	These CAR-T cells will not be affected in the process of lymphocyte clearance, and have a survival advantage compared with T cells in patients (35)
**2023**	CD19, CD47	ALL	B2M, CIITA, and TRAC	CRISPR/Cas9-mediated B2M, CIITA, and TRAC knockout	Avoiding the occurrence of graft-versus-host disease (GvHD) and improving the persistence and proliferation of these CAR-T cells (36)
**2021**	Her2	breast cancer	A_2A_R	CRISPR/Cas9-mediated A_2A_R knockout	Enhancing the function of CAR T cells and producing resistance to adenosine
**2023**	CD38	ALL	CD38	Using CRISPR/Cas9 technology to integrate CD38 CAR into CD38 locus and knock out CD38 gene simultaneously	Preventing CAR-T cells from killing each other (37)
**2023**	CD133	glioblastoma	SHP-1	CRISPR/Cas9-mediated SHP-1 knockout	Improving tumor killing ability by promoting the release of TNF-α, IL-2 and IFN-γ from CAR-T cells (38)
**2020**	mesothelin	solid tumors	TGFBR2	CRISPR/Cas9-mediated TGFBR2 knockout	Improving the durability and proliferation of CAR-T cells (39)
**2023**	CD7	ALL	CD7	Using CRISPR/Cas9 technology to integrate CD7 CAR into CD7 locus and knock out CD7 gene simultaneously	Reducing the cannibalism of CAR-T cells and enhancing tumor rejection (40)

NHL, Non-Hodgkin Lymphoma; ALL, Acute Lymphocytic Leukemia; MM, Multiple Myeloma; SOCS1, suppressor of cytokine signaling 1 MSLN, mesothelin LAG-3, lymphocyte activation gene-3; TNBC, triple-negative breast cancer; TRAC, T-cell receptor alpha constant; B2M, beta-2 microglobulin; EGFRvIII, Epidermal growth factor receptor variant III; TRAC, T-cell receptor α constant; DGK, diacylglycerol kinase; GM-CSF, granulocyte-macrophage colony-stimulating factor; CRS, cytokine release syndrome; sCRS, severe cytokine release syndrome; CIITA, MHC class II transactivator; A2AR, Adenosine receptor A2A; TGFBR2, TGF-β receptor II.

Currently, the majority of extensively researched immune checkpoints are situated on the surface of cells. However, scientists have also identified certain checkpoints within cells, including phosphatase 1B (PTP1B). The utilization of CRISPR/Cas9 technology to eliminate PTPN1 has the potential to induce STAT5 signal activation, thereby facilitating the proliferation and activation of T cells (preclinical study) (41). Hence, the utilization of CRISPR/Cas9 technology to eliminate extracellular or intracellular immune checkpoints has the potential to augment the activation of CAR-T cells and bolster their antitumor efficacy. Banta et al. (42) discovered that the simultaneous inhibition of PD-1 and TIGIT leads to a more pronounced restoration of CD226 co-activation signaling compared to single inhibition, resulting in enhanced immune activity of CD8+ T cells (preclinical study). The finding establishes a theoretical foundation for the utilization of dual blockade of PD-1 and TIGIT in cancer immunotherapy. Moreover, some clinical trials have demonstrated the feasibility of utilizing multi-site CRISPR-Cas9 edited engineered T cells in humans (24, 43). This finding provides a theoretical basis for the application of CRISPR/Cas9 technology in knocking out multiple immune checkpoints and achieving efficient editing efficiency.

#### 2.1.2 Enhancing CAR-T cell resistance to exhaustion

A significant challenge encountered in cancer treatment with CAR-T cell therapy pertains to the persistence of CAR-T cells. The persistence exhibits a positive correlation with the anti-tumor effect (44, 45), and plays a crucial role in preventing tumor recurrence. However, the duration of CAR-T cell activity and functionality within the body is constrained. Enhancing the survival duration of CAR-T cells and augmenting the survival rate can effectively enhance their long-term effectiveness. Several factors affect the persistence of CAR-T cells *in vivo*, including the structure of CARs, the quality and differentiation status of T cells, the exhaustion of CAR-T cells, and the differentiation phenotype of CAR-T cells (46–49).

T cell exhaustion refers to the progressive decline in the effector function of T cells as a result of long-term exposure to persistent antigens or chronic inflammation. Persistent antigen exposure will lead to the depletion of CAR-T cells and promote the transformation of CD8^+^ T cells into NK-like T cells (50). The exhaustion of CAR-T cells is a significant hindrance to their effectiveness in the treatment of solid tumors. CAR-T cells in an exhausted state are typically characterized by high expression of their immune checkpoints, including PD-1, TIM-3, and CTLA-4 (46, 51). By inhibiting these immune checkpoints, the exhaustion of CAR-T cells can be mitigated (18). Agarwal et al. (21) employed CRISPR/Cas9 technology to delete CTLA-4 in CAR-T cells, resulting in enhanced proliferation, persistence, and cytolytic activity of CAR-T cells (preclinical study). Moreover, the elimination of specific genes in T cells through CRISPR/Cas9 technology can also enhance the persistence of CAR-T cells. For instance, disrupting PR domain zinc finger protein 1 (PRDM1) through CRISPR/Cas9 technology ultimately enhances the persistence by promoting the expansion of memory CAR-T cells (preclinical study) (52). Furthermore, in order to prevent exhaustion, it is possible to enhance the resistance of CAR-T cells to inhibitory cytokines, in addition to targeting immune checkpoint genes and specific genes. Within the tumor microenvironment (TME), TGF-β serves as a crucial inhibitory cytokine. When TGF-β binds to TGF-β receptor II (TGβRII), it hinders the functionality of T cells (53). Alishah et al. (54) utilized CRISPR/Cas9 technology to knock out the TGFβRII gene in CAR-T cells, resulting in improved resistance to exhaustion and enhanced durability of these cells (preclinical study).

To mitigate the exhaustion of CAR-T cells, it is proposed to disrupt specific regulatory factors associated with T cell exhaustion. A study showed that the transcription factors ID3 and Sox4 were elevated in CAR-T cell dysfunction and exerted control over exhaustion-related genes (50). Employing CRISPR/Cas9 technology, researchers successfully knocked out ID3 and Sox4 in T cells, subsequently constructing CAR-T cells. The outcomes demonstrated that the downregulation of ID3 and SOX4 expression considerably postponed CAR-T cell dysfunction and enhanced their tumor-killing ability (preclinical study) (50). Furthermore, the incorporation of cytokine genes into CAR-T cells has been demonstrated to enhance the durability of CAR-T cells. The development of fourth-generation CAR-T cells builds upon the foundation of second or third-generation CAR-T cells by introducing additional anti-tumor cytokine receptor genes within the intracellular domain. Notably, research has indicated that various anti-tumor cytokines, including IL-7 (55), IL-12 (56), IL-15 (57), IL-18 (58), and IL-21 (59), can augment the functionality of CAR-T cells. Consequently, the utilization of fourth-generation CAR-T cells engineered with these cytokines can further enhance the persistence, proliferation, and cytotoxicity of CAR-T cells. CRISPR/Cas9 technology can more easily and accurately realize the construction of the fourth generation CAR sequence.

Enhancing the resistance of CAR-T cells to exhaustion holds significant importance in augmenting treatment durability, ameliorating treatment response rates, mitigating the risk of tumor recurrence, and facilitating the implementation of individualized treatment strategies. Presently, extensive research is being conducted on the persistence of CAR-T cells. The advent of CRISPR/Cas9 technology has furnished a precise and efficient instrument for optimizing and enhancing CAR-T cells. Nevertheless, additional research and experimentation are necessary to assess the effect of CRISPR/Cas9 technology on enhancing the anti-exhaustion ability of CAR-T cells, thereby promoting their clinical transformation.

#### 2.1.3 Assisting in the development of universal CAR-T cells

Currently, the predominant form of CAR-T cells employed in clinical therapy is autologous CAR-T cells, wherein the primary source of T cells is derived from the patients themselves. This approach offers the advantage of circumventing the risk of immune rejection. However, autologous CAR-T cells are not without their drawbacks, such as the protracted duration required for their manufacturing to achieve a ready-to-use state, the limited quantity and quality of T cells contingent upon the patients’ health status, and the substantial financial burden incurred. Of utmost significance is the necessity to repeat the long and costly process of CAR-T cell manufacturing should the target tumor antigens undergo mutation or loss. To address the scarcity of autologous CAR-T cells, scholars conducted research and clinical experiments on allogeneic CAR-T cells utilizing CRISPR/Cas9 technology (60). Allogeneic T cell-derived CAR-T cells, also known as off-the-shelf or universal CAR-T cells, were developed as a solution to the shortage of autologous CAR-T cells. Nonetheless, the primary drawback of universal CAR-T cells lies in their potential to induce graft-versus-host disease (GvHD) and host-versus-graft response (HvGR). A comparison between autologous CAR-T cells and universal CAR-T cells is presented in **Table 3**.

**Table 3:** Comparison between autologous CAR-T cells and universal CAR-T cells.

Feature	Autologous CAR-T Cells	Universal CAR-T Cells
**T Cell Source**	From the patients	From healthy people
**T Cell Status**	Limited by the patient’s health condition, difficult to ensure optimal status	Sourced from healthy individuals, T cells in good condition
**Treatment Applicability**	Personalized treatment, applicable to the cancer patient himself	Broad, applicable to various cancer patients
**Immunological Tolerance**	High	May induce immune rejection
**Risk of Immune Rejection**	Low	High
**Manufacturing Complexity**	Relatively simple	Relatively complex
**Time from Manufacturing to Treatment**	Long, approximately 2 weeks to 1 month	Very short
**Treatment Cost**	High	Relatively low
**Persistence**	Long-lasting	May be shorter
**Potential Side Effects**	Cytokine release syndrome, neurotoxicity, etc.	In addition to cytokine release syndrome and neurotoxicity, graft-versus-host disease (GVHD) may also occur
**Development Stage**	Used in clinical treatment for hematological malignancies	A number of allogeneic therapies have been in the clinical trial stage
**Treatment Indications**	Hematological malignancies and solid tumors	Hematological malignancies and solid tumors
**Treatment Response Monitoring**	Requires close monitoring and management	Requires close monitoring and management
**Follow-up Management**	Requires long-term follow-up and monitoring	Requires long-term follow-up and monitoring

The researchers found that autologous CAR-T cells demonstrated superior efficacy, persistence, and reduced side effects compared to allogeneic CAR-T cells (61). Therefore, in recent times, researchers have continuously optimized the allogeneic CAR-T cells. For instance, Li et al. (62) employed CRISPR/Cas9 technology to eliminate T-cell receptor (TCR) and human leucocyte antigen (HLA)-I/II genes from CAR-T cells, while incorporating exogenous expression of HLA-E (preclinical study). The findings demonstrated that this approach effectively prevented rejection and enhanced the durability of CAR-T cells. Furthermore, an alternative approach for the production of an inexhaustible reservoir of universal allogeneic CAR-T cells for cancer immunotherapy involves the utilization of induced pluripotent stem cells (iPSCs) as raw materials. This method holds considerable appeal due to the inherent characteristics of iPSCs, which possess limitless proliferative capacity and the capability to differentiate into various cell lineages in the body. Iriguchi et al. (63) demonstrated that iPSCs can serve as a source for generating mature T cells, which can be utilized for off-the-shelf T cell immunotherapy (preclinical study). Ensuring the robust proliferative capacity of these iPSCs-derived T cells is crucial, and the researchers observed an approximately 200-fold expansion of these T cells during subsequent culture. Wang et al. (64) employed CRISPR/Cas9 technology to integrate the CAR gene into the endogenous TCRα constant (TRAC) locus chain, resulting in the successful development of CAR-T cells with diminished immunogenicity derived from iPSCs (preclinical study). The findings demonstrate that these iPSCs-derived CAR-T cells exhibit enhanced tumor cytotoxicity, prolonged survival duration, and decreased likelihood of eliciting an allogeneic response in the host. In addition, it is also feasible to modify some genes with CRISPR/Cas9 technology to enhance the therapeutic effect of iPSCs-derived CAR-T cells. Ueda et al. (65) successfully optimized iPSC-derived CAR-T cells through gene modification using CRISPR/Cas9 technology (preclinical study). Specifically, they achieved enhanced proliferation capacity and longevity of these CAR-T cells by disrupting the diacylglycerol kinase gene and introducing transduced interleukin-15 (IL-15) and its receptor subunit (IL-15Rα) gene expression.

Presently, the investigation into universal CAR-T cells is in its nascent phase, necessitating additional research and clinical trials to validate their viability and efficacy. Notably, 35 allogeneic CAR-T cell-related clinical trials (Phase I- Phase II) are ongoing (66). Undeniably, the utilization of CRISPR/Cas9 technology offers a crucial means and approach for the development and further optimization of universal CAR-T cells.

### 2.2 CRISPR/Cas9 editing technology can reduce the manufacturing costs of CAR-T cells

CAR-T cell therapy is limited in its clinical application to some extent by its high cost. This is mainly due to the complicated manufacturing process of CAR-T cells and the high cost of raw materials. Although there are options for automated manufacturing of CAR-T cells such as the CliniMacs Prodigy and Lonza Cocoon system, manual and modular methods are still largely used by CAR-T developers which inevitably contributes to increased production cost (14). CAR-T cell manufacturing costs can be reduced by 1) further optimizing CAR-T cell manufacturing and production processes to improve production efficiency and yield; and 2) establishing strict quality control systems to ensure CAR-T cell quality and consistency, reducing unnecessary waste and duplication.

The manufacturing cycle of CAR-T cells is usually about two weeks. To expedite this process, researchers have employed the transduction of non-activated resting T cells with lentiviral vectors. This enables the direct conversion of extracted T cells from patients into CAR-T cells, resulting in a more rapid manufacturing time. Under the condition that the function was not affected, the manufacturing time could be shortened to 24 hours (67). Furthermore, novel CAR-T cell manufacturing platforms can also shorten CAR-T cell manufacturing time. The Novartis T-charge platform represents an advanced platform of CAR-T cell therapy manufacturing, with the primary objective of reducing *in vitro* culture time and enhancing T cell proliferation potential. Dickinson et al. (68) utilized the T-charge platform to generate a novel autologous CD19-CAR-T cell therapy (YTB323), which shared the same CAR construct as tisagenleucel (clinical phase I/II). Notably, the findings demonstrated that YTB323 could be manufactured within 2 days, while preserving T cell stemness, and exhibited enhanced *in vivo* expansion and anti-tumor efficacy at a lower dose. The FasTCAR platform of Gracell Biotechnologies can shorten the CAR-T manufacturing time from two weeks to one day. Currently, there are ongoing clinical trials (NCT04638270, NCT05840107, and NCT04935580) evaluating the efficacy and safety of CAR-T cells produced using the FasTCAR platform. Moreover, The CRISPR/Cas9 technology exhibits high efficacy in the editing of genes and is capable of achieving precise modifications within a comparatively brief timeframe. Zhang et al. (22) used CRISPR/Cas9 technology to target the target sequence (clinical phase I). They introduced the CAR sequence template by electroporation, and inserted the CAR sequence into the target gene by homologous recombination repair mechanism, thus producing CAR-T cells independent of the traditional viral based transduction approach. This method simplifies the manufacturing process and shortens the manufacturing time. These methods reduce manufacturing costs indirectly by shortening the manufacturing time.

It should be pointed out that CRISPR/Cas9 editing technology itself cannot directly reduce the manufacturing cost of CAR-T cells. Because the technology is mainly used for gene editing, it does not directly affect other cost factors in the CAR-T cell manufacturing process. The cost of CAR-T cell p manufacturing involves many aspects, including cell culture, quality control, equipment, and manpower. CRISPR/Cas9 editing technology can indirectly reduce the cost of CAR-T cell manufacturing. Firstly, the utilization of CRISPR/Cas9 technology has the potential to enhance the functionality and activity of CAR-T cells, thereby augmenting their anti-tumor effectiveness. This, in turn, can lead to a reduction in the dose and duration of subsequent treatments, consequently mitigating the financial burden associated with therapy. Secondly, the application of CRISPR/Cas9 technology can enhance the proliferative and survival capabilities of CAR-T cells by modifying genes associated with cellular expansion and persistence. This can shorten the expansion time of CAR-T cells and reduce the culture costs. To summarize, the utilization of CRISPR/Cas9 technology has the potential to indirectly mitigate the expenses associated with the production of CAR-T cells by optimizing the function of CAR-T cells, enhancing their proliferation and persistence.

### 2.3 CRISPR/Cas9 technology faces safety issues in the CAR-T cell field

Despite the fact that CRISPR/Cas9 technology is an effective gene editing tool, there are still some safety problems to be solved in its application. These concerns encompass off-target effects, DNA damage, and immunogenicity (69–71). Some studies have demonstrated that the utilization of CRISPR technology for gene editing in human embryos or cells frequently results in the elimination of substantial segments of chromosomes (72–74). The employment of this technology also has the potential to activate p53, leading to DNA damage, while the activated p53 subsequently hampers the efficiency of the CRISPR-Cas9 system for gene editing (70, 75). Investigations involving the editing of human embryonic stem cells and other cell types have revealed that the Cas9 protein can directly interact with the DNA-PK complex, impeding the DNA repair pathway and potentially heightening the risk of chromosome fragmentation (76). Moreover, the utilization of the CRISPR-Cas system has the potential to induce unforeseen structural variations (SVs), including deletions, duplications, and insertions (77). Consequently, CRISPR/Cas9 technology may raise some potential safety issues for CAR-T cell therapy that need to be carefully considered and addressed.

June et al. (43) proved for the first time in a phase I clinical trial that it was feasible to modify T cells with CRISPR/Cas9 technology. The researchers used CRISPR-Cas9 to knock out the PD-1 gene and two genes encoding the T cell receptor (TCR) (TRAC and TRBC) of T cells. The outcomes revealed that the three patients who received T cells engineered with CRISPR-Cas9 did not exhibit cytokine release syndrome (CRS), while the modified T cells exhibited enduring survival and expansion. Hamilton et al. (78) highly praised this research and put forward some thoughts in this field, including 1) it is not clear whether the cells edited by Cas9 are immunogenic; 2) Whether the residual Cas9 will trigger the immune response; 3) Whether the engineered T cells edited by CRISPR are effective for advanced cancer. Furthermore, genome editing by CRISPR/Cas9 has the potential to induce chromosomal deletions and cause chromosomal instability in the preclinical production of CAR-T cells (79, 80). Tsuchida et al. (80) discovered that the utilization of CRISPR technology to disrupt the TRAC gene results in a range of 4%-22% of T cells exhibiting significant chromosome deletion (clinical phase I). To ascertain the generalizability of this occurrence, the investigators conducted CRISPR screening using a gRNA library encompassing all chromosomes and subsequently analyzed the chromosome deletions through experimental means. The findings indicate that 55% of gRNA sequences induce chromosome deletion, and any chromosome is susceptible to substantial fragment or complete chromosome deletion as a consequence of gene editing. Moreover, gRNA sequences located in proximity to the centromere exhibit a higher propensity for chromosome deletion. Finally, the researchers revealed that DNA double strand breaks (DSB) caused by CRISPR gene editing and the expression level of p53 in cells were the key factors leading to chromosome deletion. They tweaked the CAR-T cell manufacturing process by first performing Cas9-mediated gene editing on T cells and then activating those T cells. This method effectively reduces the frequency of chromosome deletions. Furthermore, the utilization of the CRISPR system may result in the enrichment of p53 mutant cells, thereby potentially instigating the progression of cancer (75). Jiang et al. found that transient inhibition of p53 may be a new strategy to reduce the enrichment of mutant cells (81).

Despite the safety concerns associated with its application, CRISPR/Cas9 technology undeniably holds significant potential for optimizing and enhancing CAR-T cell therapy. To address these safety issues, scientists and researchers are actively refining CRISPR-Cas9 technology and conducting meticulous preclinical and clinical trials to assess its safety and efficacy. In 2023, the Food and Drug Administration (FDA) approved the inaugural gene editing therapy, known as Casgevy, utilizing the CRISPR technology. Casgevy represents an *in vitro* gene editing therapy that employs CRISPR/Cas9 genome editing to alter the hematopoietic stem cells of patients, thereby offering a potential treatment for sickle cell disease. This significant achievement marks a noteworthy advancement in the field of biotechnology. When the BCL11A gene undergoes mutation, certain adults have the ability to generate fetal hemoglobin. Casgevy conducted a simulation of this mutation by employing gene editing technology to precisely cleave the BCL11A gene in hematopoietic stem cells of patients, thereby inducing the release of fetal hemoglobin (82). Thus far, certain phase I clinical trials have substantiated the safety and feasibility of utilizing CRISPR/Cas9-based CAR-T cells in human trials (22, 24, 35, 83).

## 3 Conclusion and prospect

The CRISPR/Cas9 technology, possessing immense potential, has proven to be highly beneficial in the realm of life sciences. It provides an important tool for researchers and clinicians in this field to carry out innovative research and develop treatment methods. Moreover, the CRISPR/Cas9 technology holds significant importance in the advancement of CAR-T cell optimization. The initial demonstration of CRISPR/Cas9 technology in 2012 showcased its potential for gene editing in prokaryotes, and subsequent studies confirmed its efficacy in gene editing in human and mammalian cells (15, 16). After 2012, CAR-T cell technology began to develop rapidly. Eventually, these two major technologies formed a robust alliance. In recent years, this strategy has been favored by experts in the CAR-T cell field and achieved rapid development. The application of CRISPR/Cas9 technology in CAR-T cell therapy holds significant potential. Additionally, more advanced CRISPR/Cas systems, such as Cas13, Cas14, Cas12a (Cpf1), and nickase Cas9 (nCas9), may be more efficient, more accurate, or safer than Cas9 system in gene editing (84, 85). For instance, Cas13system can directly edit single-stranded RNA without transcribing RNA into DNA, which simplifies the editing process and has high editing efficiency and specificity (86). In comparison to Cas9 and Cas13system, Cas14 system possesses a smaller size, facilitates easier transfer and expression within cells, and demonstrates enhanced specificity in gene editing (87, 88). These CRISPR/Cas systems may help to overcome the current obstacles and barriers faced by CAR-T cells, and may pave the way for a substantial breakthrough in treating solid tumors with CAR-T cells in the future.

## Author contributions

RT: Conceptualization, Data curation, Investigation, Methodology, Project administration, Writing – original draft, Writing – review & editing. XB: Investigation, Software, Visualization, Writing – original draft. XH: Data curation, Investigation, Visualization, Writing – original draft. JY: Investigation, Methodology, Writing – original draft. YM: Investigation, Validation, Writing – original draft. WC: Investigation, Writing – original draft. DZ: Investigation, Writing – original draft. ZL: Conceptualization, Resources, Supervision, Writing – review & editing.
